# Differentiation of primary central nervous system lymphoma from glioblastoma using optical coherence tomography based on attention ResNet

**DOI:** 10.1117/1.NPh.9.1.015005

**Published:** 2022-03-23

**Authors:** Sanford P. C. Hsu, Tien-Yu Hsiao, Li-Chieh Pai, Chia-Wei Sun

**Affiliations:** a Taipei Veterans General Hospital, Department of Rehabilitation and Technical Aid Center, Taipei, Taiwan; b Taipei Veterans General Hospital, Neurological Institute, Division of General Neurosurgery, Taipei, Taiwan; c National Yang Ming Chiao Tung University, School of Medicine, Taipei, Taiwan; d National Yang Ming Chiao Tung University, College of Electrical and Computer Engineering, Department of Photonics, Hsinchu, Taiwan

**Keywords:** glioblastoma, primary central nervous system lymphoma, optical coherence tomography, convolutional neural network

## Abstract

**Significance:** Differentiation of primary central nervous system lymphoma from glioblastoma is clinically crucial to minimize the risk of treatments, but current imaging modalities often misclassify glioblastoma and lymphoma. Therefore, there is a need for methods to achieve high differentiation power intraoperatively.

**Aim:** The aim is to develop and corroborate a method of classifying normal brain tissue, glioblastoma, and lymphoma using optical coherence tomography with deep learning algorithm in an *ex vivo* experimental design.

**Approach:** We collected tumor specimens from ordinal surgical operations and measured them with optical coherence tomography. An attention ResNet deep learning model was utilized to differentiate glioblastoma and lymphoma from normal brain tissues.

**Results:** Our model demonstrated a robust classification power of detecting tumoral tissues from normal tissues and moderate discrimination between lymphoma and glioblastoma. Moreover, our results showed good consistency with the previous histological findings in the pathological manifestation of lymphoma, and this could be important from the aspect of future clinical practice.

**Conclusion:** We proposed and demonstrated a quantitative approach to distinguish different brain tumor types. Using our method, both neoplasms can be identified and classified with high accuracy. Hopefully, the proposed method can finally assist surgeons with decision-making intraoperatively.

## 1 Introduction

Brain tumors, whether benign or malignant, can result in increases in intracranial pressure, extrude room of normal brain tissues, and finally threaten human longevity and quality of life; therefore, effective treatments for intracranial lesions are required to render promising prognoses. Noted that properly designed therapies should be given corresponding to specific tumor types so as to bring out the desired outcome. Glioblastoma (GBM) is the most common type of malignant neoplasm and constitutes half of such tumors.^1^ According to the statistical report of the Central Brain Tumor Registry of the United States (CBTRUS), the 5-year survival rate is <8%.^1^ As a full cure of GBM is not reachable, the current mainstay of GBM treatment is to undergo maximal possible safe resection followed by adjuvant chemoradiotherapy to prolong the survival of patients.^2^^–^^4^ In addition to GBM, another malignant intracranial neoplasm primary central nervous system lymphoma (PCNSL) happened in 1.9% of brain tumors, and its 5-year survival rate is 37.6%.^1^ Nowadays, the most effective treatments of PCNSL are chemotherapy and radiotherapy.^5^^,^^6^ Usually, operational resections are discouraged as therapeutic regimens of PCNSL.^7^^,^^8^ To this end, distinguishing PCNSL from GBM has garnered attention from the perspective of clinical practice.

Owing to the differences in their radiological morphology, preoperative magnetic resonance imaging (MRI) can help to differentiate PCNSL from GBM. Research has shown that GBM displays significant imaging heterogeneity across patients, and typically, MRIs of GBM show necrosis as an important hallmark of GBM, manifesting with either rim enhancement, irregular outer shape, or solid masses.^9^^,^^10^ On the other hand, PCSNL tends to be less diffusely infiltrative compared to GBM and rarely displays a necrotic area.^11^^,^^12^ However, atypical cases can mimic one another, e.g., GBM without visible necrosis or PCNSL with evident necrosis, and this makes differentiation by gross visual inspection via conventional MRI difficult. In addition to conventional MRI, since GBM and PCSNL are different in their functional expressions, functional MRI can help us tell their differences. Differentiation of PCNSL and GBM can be addressed based on three functional characteristics,^13^^,^^14^ and those are tumor vascularity,^15^ vascular permeability,^16^^,^^17^ and tumor cellularity.^18^^,^^19^ As PCNSL exhibits angiocentric growth patterns and lacks neoangiogenesis, both tumor blood flow and vascularity of PCNSL are less than those of GBM. The greater extent of the disruption of the blood–brain barrier in PCNSL, the higher its permeability is. Also, PCNSL usually has denser tumor cellularity than GBM. Among all functional MRI, dynamic susceptibility contrast MR perfusion demonstrated the highest specificity of PCNSL and arterial spin labeling MR perfusion for the highest sensitivity.^13^^,^^14^ Nonetheless, some of those require the use of contrast agents, and complicated sequences take considerable measuring time, which makes them unsuitable for clinical use.

The gold standard of diagnosis nowadays is the paraffin section, for which a sample is extracted intraoperatively. In spite of the microscopic resolution enabling paraffin to distinguish PCSNL from GBM, the procedure of making a paraffin section takes days to obtain its histological outcome and thus is not applicable for intraoperative diagnosis. As a substitute for a paraffin section, the frozen section provides acceptable classification power of brain tumor categories and thus is used in the standard examination procedure intraoperatively. Unfortunately, owing to the intrinsic limitations of the frozen section,^20^^,^^21^ its sensitivity for PCNSL is still poor.^22^ A microscopic examination of intact tissue morphology is needed to achieve high differentiation power. Optical coherence tomography (OCT) can permit real-time and depth-resolved images with submicron resolution, and therefore OCT is a good tool in the sense of surgical applicability.

Research using OCT for brain tumor detection have shown that normal brain tissues are structureless and appear homogeneous in conventional OCT images whereas GBM tends to be more heterogeneous and displays microstructures such as microcysts, calcification, and hemorrhaging.^23^^–^^27^ According to Böhringer’s research,^23^ morphological features, including nonuniform attenuation and pathological microstructure, can distinguish high-grade gliomas from normal tissues. Yet, the presence of microstructure was not quantified and thus subjective. Notwithstanding that other groups also found an abnormal low attenuation coefficient of GBM,^28^^,^^29^ the calculation of the attenuation coefficient is restricted to images that do not exhibit microstructures, which are very common in GBM.^30^ A similar scenario also happened in distinguishing melanoma from benign nevus in dermatology. Turani et al. developed a patented image analysis algorithm called optical properties extraction to extract optical radiomic signatures from OCT images.^31^ They combined the extracted morphological features, including mean and standard deviation of scattering and absorption coefficients, and the mean of the anisotropy factor, with machine learning models to improve the identification of melanoma. Finally, a superb differentiation power between early malignant melanoma and benign nevus was achieved, showing the potential of machine learning in lesion classification using morphological features. Here, to quantitatively differentiate GBM and PCNSL from normal tissues via morphological features in a more automatic way, we manage to utilize a convolutional neural network for classification in this study. Lately, the preliminary results of classifying infiltrative gliomas were reported^32^ in an international conference and proved the applicability of convolutional neural networks for brain tumor classification. Yet, so far there is still no report observing PCNSL using OCT, which is also one of our aims in this study.

## 2 Methods

**Fig. 1:**
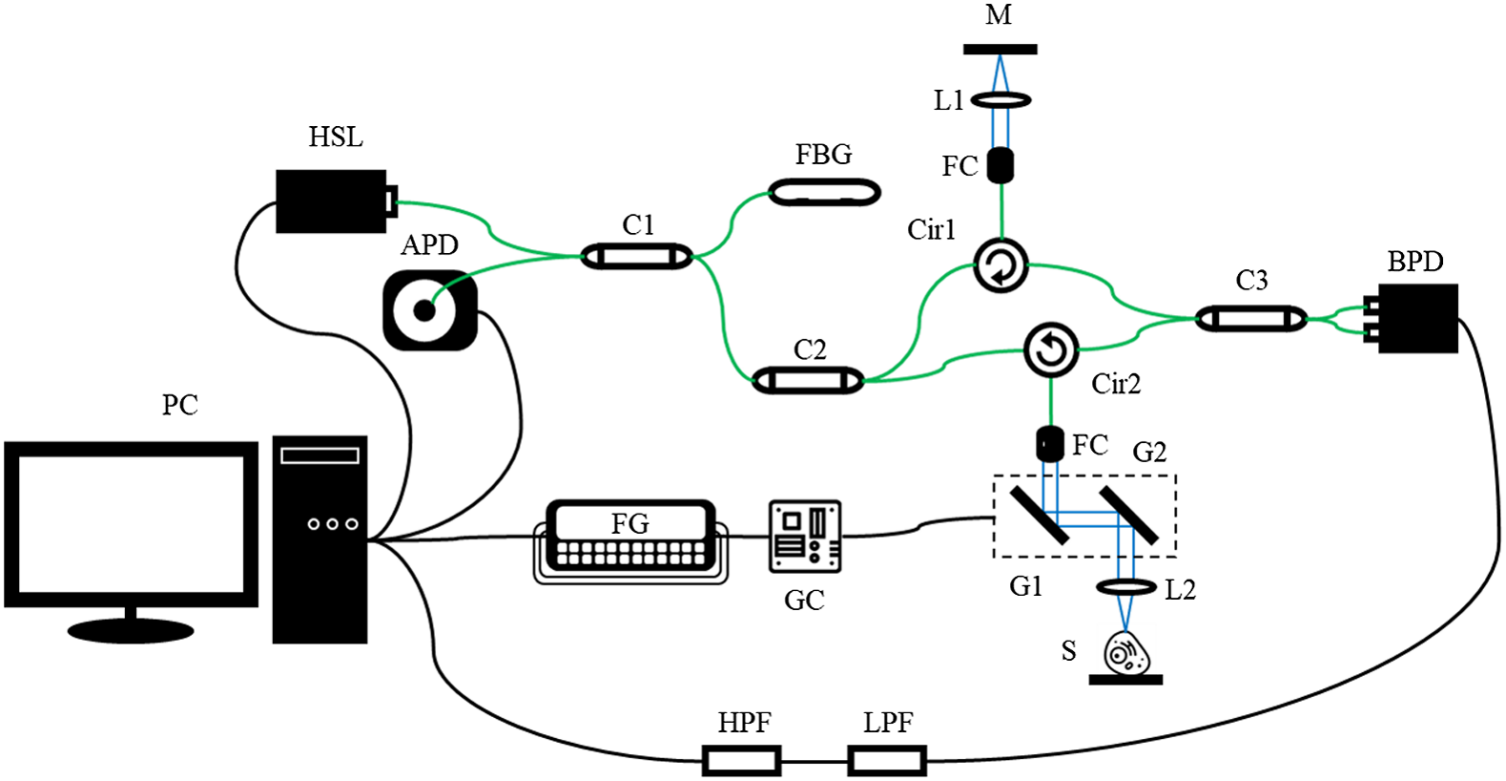
The schematic design of the swept-source OCT system. Black curves, green curves, and spaced blue curves represent electrical wires, optical fiber transmission, and air-space beam transmission, respectively. APD, amplified photodetector; BPD, balanced photodetector; C1, 1:99 coupler; C2, 20:80 coupler; C3, 50:50 coupler; Cir1, Cir 2, circulator; FBG, fiber Bragg grating; FC, fiber coupler; FG, function generator; G1, G2, Galvano scanner; GC, Galvano controller; HPF, high-pass filter; HSL, high-speed laser; L1, L2, lens; LPF, low-pass filter; M, mirror; PC, personal computer; S, sample.

In this study, we preliminarily conducted our experiment on *ex vivo* specimens. Both GBM and PCNSL samples were excised from ordinal surgical operations. Since we could not obtain normal brain tissues from surgical routines, we selected the porcine brain as a surrogate for human normal brain tissues as the porcine brain has a resemblance with human’s in histological characteristics and was used for preliminary experiments prior to clinical trials.^33^^,^^34^ After we measured specimens using OCT, the deep learning model was trained for classifications of the three categories. The results were evaluated using confusion matrix, gradient class activation mapping (grad-CAM),^35^ and t-distributed stochastic neighbor embedding (t-SNE) scatter plots.^36^ Experimental details are illustrated in the following sections accordingly (Fig. 1).

### 2.1 Sample Preparation

The recruitment was done in the Department of Neurosurgery, Taipei Veterans General Hospital, Taiwan, and written informed consent were obtained from all subjects. We recruited patients who were 20 to 65 years old and needed to undergo resection surgeries, and patients who were with metastatic brain tumors or have been treated with chemotherapy or radiotherapy were excluded. Tumor specimens were collected via lesion removal from ordinal surgical operations, and sequentially, specimens including porcine samples were preserved in formalin solution. The study was approved by the Institutional Review Board (IRB) of Taipei Veteran General Hospital (2019-07-022CC) and of National Chiao Tung University (NCTU-REC-108-066E).

### 2.2 Experimental Setup

As published beforehand,^37^ the same OCT system in our laboratory was employed in this research. A single-mode fiber-based Mach–Zehnder interferometer configuration was utilized in our OCT system design. The wavelength of the high-speed swept-source laser (HSL-20-50, Santec Corp.) was centered at 1.31  μm with a full-width at half-maximum of 100 nm, outputting a 15-mW optical power on average. As a consequence, the theoretical axial resolution was 8  μm in the air. The A-line scanning rate of 50 kHz was provided by the amplified photodetector (PDA05CF2, Thorlabs) on detection of the reflection from the fiber Bragg grating (FBG-SMF-1266-80-0.2-A-(2)60F/E, L=1M, Tatsuta Electric Wire & Cable Co., Ltd.) at the wavelength of 1266.0 nm and triggered the start of an A-line data acquisition. The k-linearity calibration was performed by utilizing the built-in k-trigger of the laser.

The C1 coupler separated the input light by 1:99, and the minor portion of the beam was redirected to the FBG, leading to 80% of the incoming beam reflected at the wavelength of 1266.0 nm. The APD captured the reflected beam as the A-trigger signal afterward. As for the major portion of the beam, the light was divided by the C2 coupler and entered the reference arm and the sample arm of the interferometer through two polarization-insensitive optical circulators (PICIR-1214-12-L-05-NE, OF-Link Communications Co., Ltd.), Cir1 and Cir2, with 20:80 ratio. The fiber collimator (F260APC-C, Thorlabs) along with the achromatic lens (AC254-030-C-ML, Thorlabs) in the reference arm collimated the light, and the gold-coated mirror reflected the beam back through the same trajectory. In the sample arm, the galvanometers along with the optical elements (FC, L2) the same as those in the reference arm were added in the optical path prior to the samples. By theory, the lateral resolution of an approximate 18  μm in the air was derived. The interference signal was formed in the C3 coupler by transmitting the beams from two arms via the circulators and detected by the balanced photodetector (PDB480-AC, Thorlabs) to acquire less contaminated signals. Overall, a 91.58-dB system sensitivity was achieved. Before electric signals entered the waveform digitizer (ATS9350, Alazar Technologies), filtering by a high-pass filter (ZFHP-0R23-S+, Mini-Circuits International) and a low-pass filter (BLP-90+, Mini-Circuits International) was applied for signal conditioning with a designated frequency band (0.23 to 81 MHz). Finally, the interference signals were sampled linear in k-space accordingly.

**Fig. 2:**
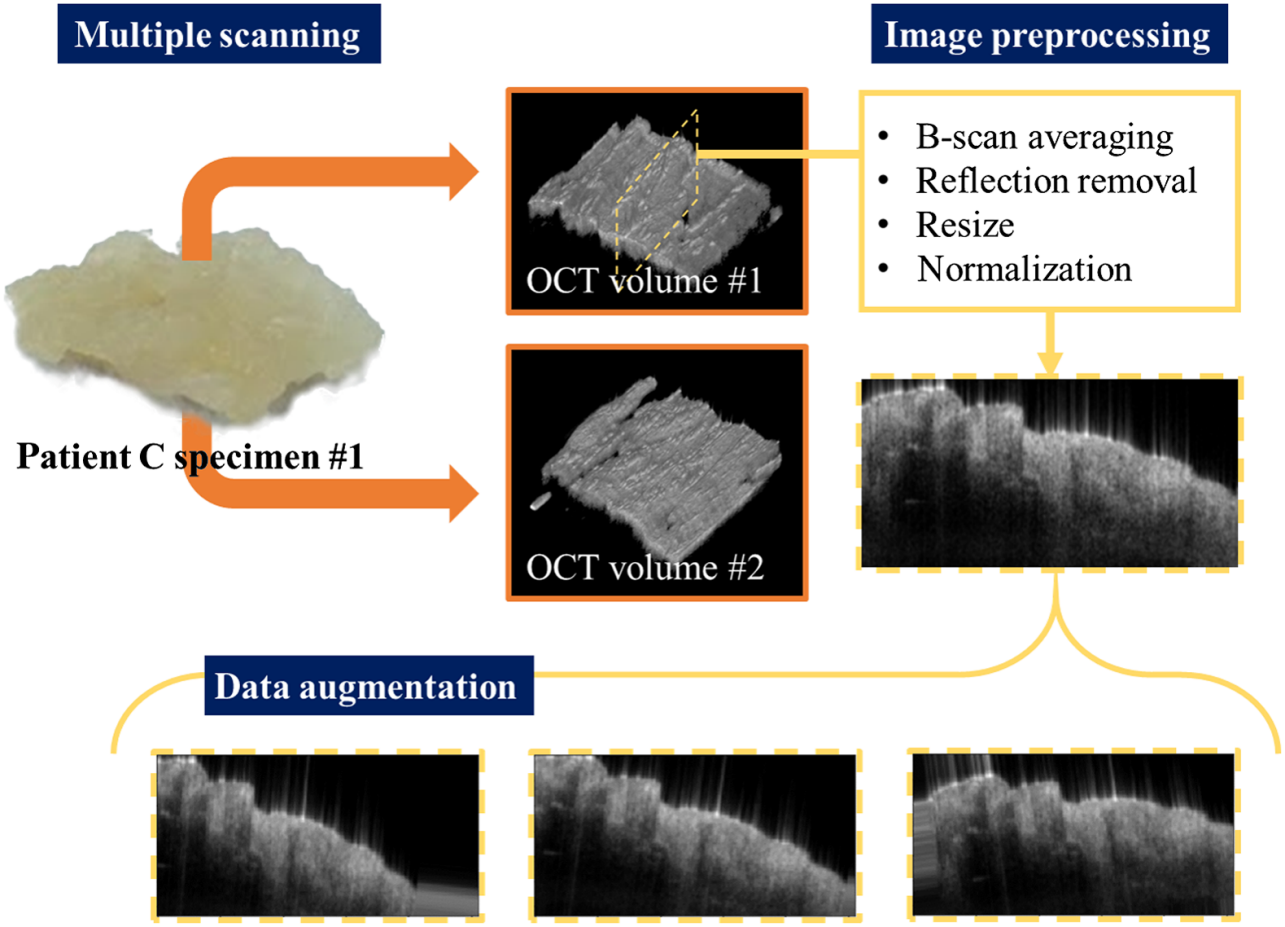
Image acquisition and processing flowchart. Multiple scans from the same specimens were acquired at different regions. The obtained images were preprocessed by B-scan averaging, reflection removal, resize, and normalization. Finally, data augmentation was employed during the model training process. The orange frames and arrows mark what is related to OCT volumetric data, while the yellow ones mark those to B-scan data.

All system controls were accomplished using the LabVIEW program. The two galvanometers (GVSM002, Thorlabs) were controlled by the function generator (AFG-2225, Good Will Instrument Co., Ltd.). Synchronized with these, the waveform digitizer performed the two-dimensional (2D) scanning. A 5  mm×5  mm scanning area was applied, in which the C-scan was made up of 1000×1000 A-scans. Each sample was measured multiple times in different scanning directions volumetrically to increase the amount of data. We changed the scanning area so each OCT volume was measured from a completely different position of a sample to minimize the structural similarity among OCT volumes obtained from the same specimens. Those frames exhibiting strong reflections were excluded from the model training because the reflections exhibited as straight striped lines, which degenerated the image quality. In this study, we aimed to preliminary verify the applicability of the proposed method, so the image quality was controlled to exclude the possible variables of outcomes. Figure 2 shows the process of image acquisition. All images were taken from different positions even from the same specimen so as to prevent our data from causing overfitting.

### 2.3 Data Processing

All data processing was done using the programming language Python v3.6 with CUDA GPU acceleration on the high-performance Windows-based computer with 16.0 GB RAM, Intel(R) Core(TM) i5-7500 CPU at 3.40 GHz, and an NVIDIA GeForce GTX1660 GPU. Despeckled images were first generated by averaging 10 adjacent B-scans after B-scans translational registered. We resized and normalized the despeckled images into the size of 128  pixels×256  pixels (physical range of 2.5  mm×5.0  mm) prior to the training to achieve the efficient training of the neural network. Data augmentation was implemented through random combinations of rotation, translation, shearing, and zooming. In the prevention of the alteration of morphological features in the OCT images, the effects of shearing and zooming were set with a ratio of 0.1.

**Fig. 3:**
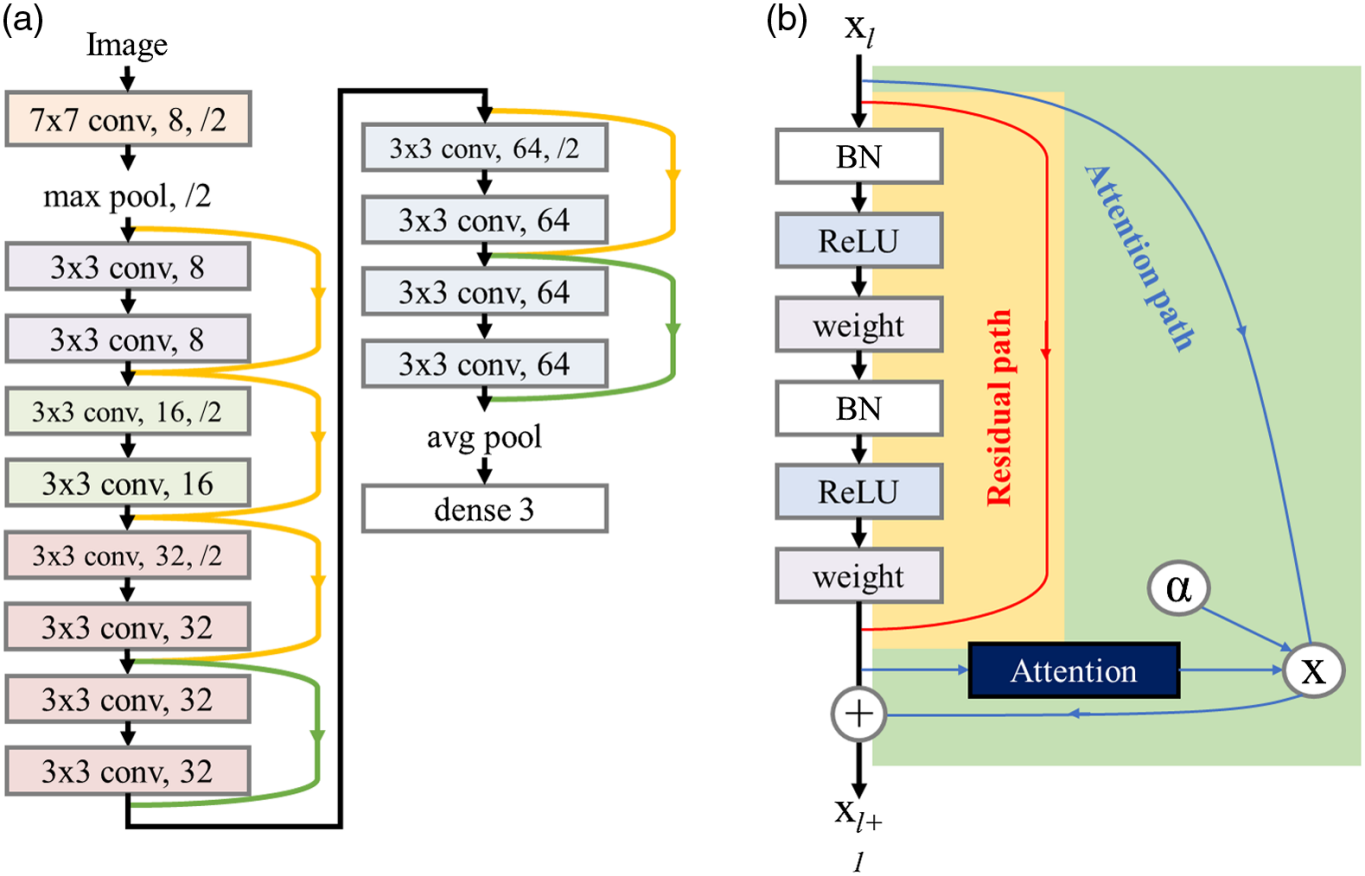
(a) The deep learning model based on modified ResNet architecture used in this study. The sidetracks represent the residual path, and the attention path was additionally applied in green sidetracks. (b) The detail scheme of a residual unit. BN, batch normalization.

Figure 3 shows our schematic design of the neural network model, namely the attention ResNet. A helpful study has shown that reordering of batch normalization, ReLU activation function, and convolution layer in the ResNet model can prevent the training process from gradient vanishing.^38^ In this study, we employed 14-layer ResNet with six improved residual units as shown in Fig. 3(b). The filter sizes, numbers, and strides (omitted if 1) of convolution layers were written in the blocks shown in Fig. 3(a), and the numbers of filters were set to be 8, 16, 32, and 64. We further applied additional attention paths on the last layers with 32 and 64 filters to detect the regional relation between high-level graphical features,^39^ and the attention paths were designed to make the network only better as long as the alpha value was larger than zero. The training was done in a batch size of 32 images, and the stochastic gradient descent optimizer was selected with a learning rate of 0.0005 and a momentum of 0.9. We also applied L2 regularization for model generalization and automatic class weights adjustment for imbalanced classes. Categorical cross-entropy was used to evaluate the performance of the optimization, and the training process terminated when the loss function of validation data no longer decreased for 10 epochs.

To inspect the model performance, two visualization techniques, grad-CAM and t-SNE, were implemented and demonstrated. Developed by R. R. Selvaraju, grad-CAM provides the mapping of which the model predicts the classifying outcome depending on those regions with higher values shown in. To be more specific, this activation map is technically derived from calculating the gradient of the output given an input image. In our application, grad-CAM was utilized to visualize what sort of features the trained model focused on. T-SNE is a powerful dimensional reduction method that retains the local structure of transformed data, and therefore t-SNE is also particularly suitable for the data representation of high dimensional data. In this research, we applied t-SNE to examine the distributions of data from different samples and their properties.

## 3 Results

### 3.1 Recruitment and Dataset

Six patients diagnosed with GBM and one with PCNSL were recruited in this study, and the specimens were extracted during the ordinal operations. To increase the amount of data, one specimen might be scanned multiple times. The recruitment detail is listed in Table 1, and NOR represented the normal brain tissue harvested from a porcine. We divided the data into the training dataset and the testing dataset, and fivefold cross-validation was utilized within the training dataset to test the model stability and reliability. All data separations were according to different OCT volumes so as to generalize the model applicability to unseen OCT images as shown in Table 2.

**Table 1:** Recruitment information.

Patient	Specimens	OCT volumes	Diagnosis
A	9	17	NOR
B	2	3	GBM
C	2	3	GBM
D	1	2	GBM
E	2	3	GBM
F	1	1	GBM
G	1	1	GBM
H	7	8	PCNSL

Note: Patient A is porcine, which is used as a surrogate of the normal human subject.

**Table 2:** Data separation.

Diagnosis	Training dataset		Testing dataset	
	OCT volumes	Frames	OCT volumes	Frames
Normal tissue	15	14,517	2	1940
Glioblastoma	10	9698	3	2912
PCNSL	5	4854	3	2913

### 3.2 Characteristics of Neoplasms

**Fig. 4:**
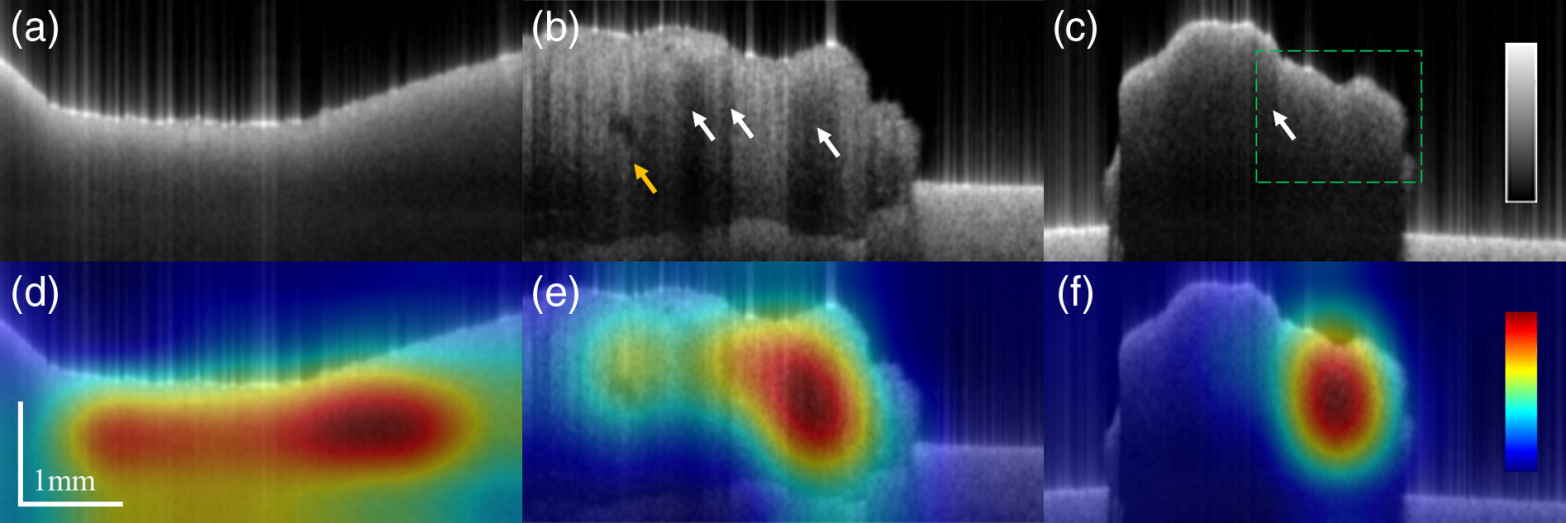
Examples of OCT intensity images (upper row) and CAM images (lower row) of [(a), (d)] normal tissues, [(b), (e)] GBM, and [(c), (f)] PCNSL. White arrows indicate nonuniform attenuation, the yellow arrow indicates the presence of the irregular microstructure, and the green dashed rectangle marks the area of the lower attenuation in PCNSL.

**Fig. 5:**
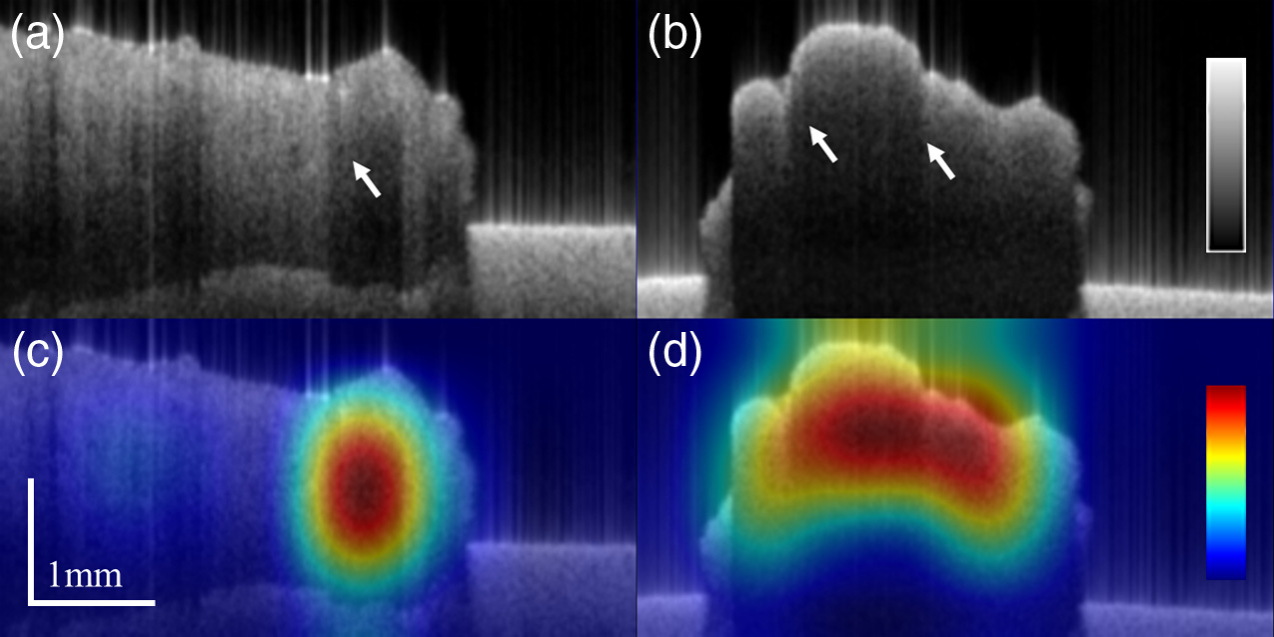
Examples of OCT intensity images (upper row) and CAM images (lower row) of [(a), (c)] misclassified GBM and [(b), (d)] PCNSL. White arrows indicate nonuniform attenuation. The misclassified GBM frames displayed structureless appearances, whereas that of PCNSL showed stronger attenuation nonuniformity.

Typical OCT images of different kinds were shown in the upper row of Fig. 4. In OCT images, normal tissue exhibited homogeneous appearances whereas GBM, in general, displayed irregular holes and abnormal attenuation as reported before.^25^ In contrast, instead of showing microstructural features, PCNSL surprisingly tended to be homogeneous with a few abnormalities of attenuation. Based on our visual inspection, one of the GBM OCT volumes appeared structureless and was partially classified as PCNSL by our model as shown in Fig. 5(a). On the other hand, the model was prone to predict PCNSL images with strong attenuation discontinuity as GBM as shown in Fig. 5(b). This evidence implied that the classification result of the model depends on tissue structural texture and attenuation continuity.

We applied the grad-CAM to visualize areas where the model focused as shown in the lower row of Fig. 4. The images were generated by combining OCT intensities and the heatmap of grad-CAM images. In normal tissues, homogeneous intensities were identified as expected, and areas were evenly focused by grad-CAM, which is quite reasonable. By contrast, nonuniform attenuations and microstructures were both identified in GBM, displaying a more concentrated grad-CAM heatmap image. Finally, in Fig. 4(c), we observed an attenuation cliff at the edge of strong and weak attenuations. On the other hand, homogeneous intensities were identified in PCNSL yet with a more concentrated heatmap in the grad-CAM image in comparison to that of the normal tissues. Figure 4(f) shows strong activation on the region with lower attenuation. Although PCNSL shows a bit of nonuniform attenuation as we have observed, the decisive feature of PCNSL by the model was instead the slowly attenuating rate of PCNSL as implied in its grad-CAM. Hereby, we speculated that what our model focused on is low attenuation, which can also be related to our visual observation.

### 3.3 Quantitative Evaluations

**Fig. 6:**
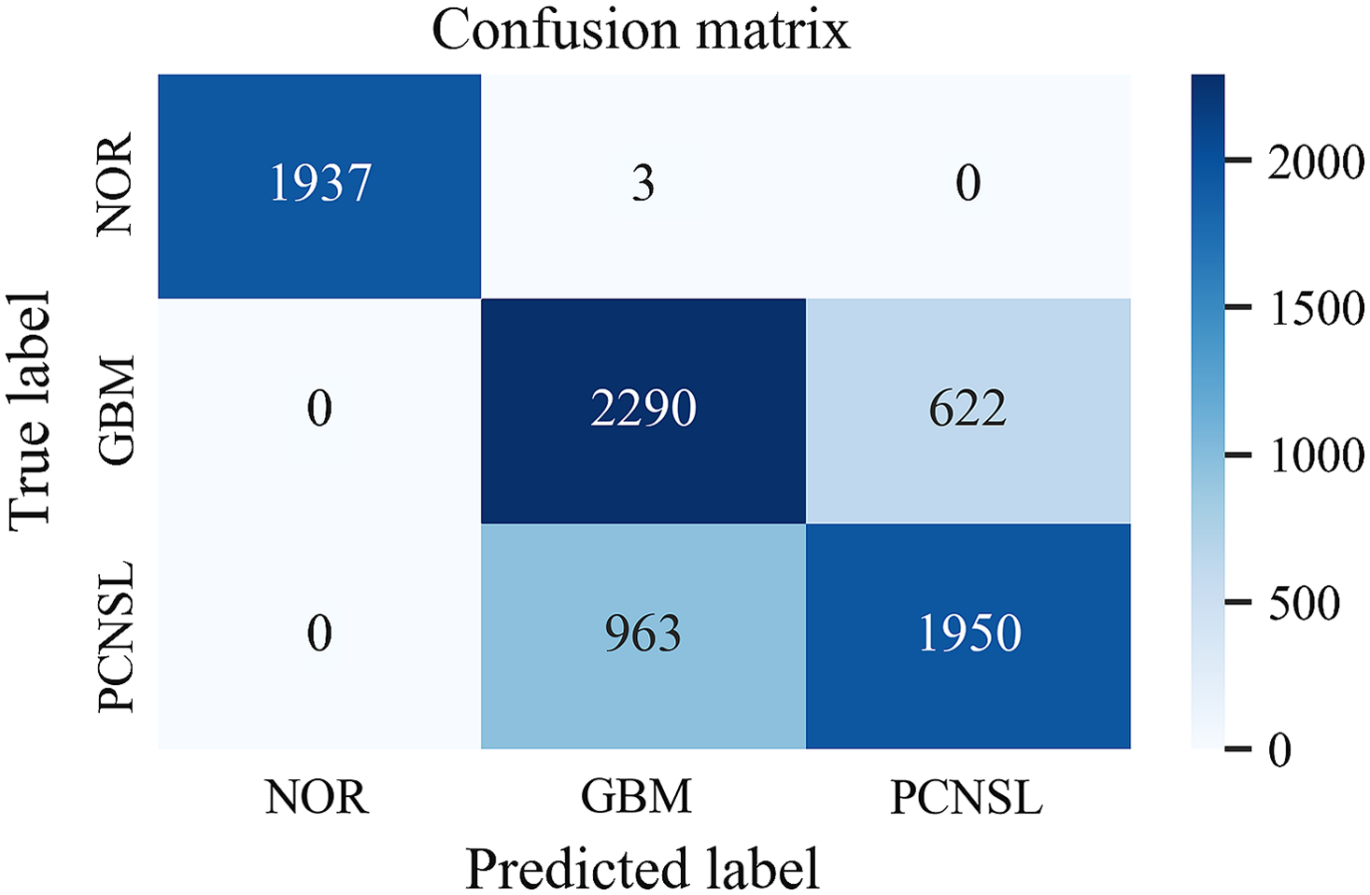
The confusion matrix of the testing data from one model. The sensitivities and specificities of GBM and PCNSL were 78.6%, 80.1%, 66.9%, and 87.2%, leading to the accuracy of 79.5%.

The accuracies of the proposed method on training data, validation data, and testing data were 97.5%, 85.8%, and 80.3% with the standard deviations of 5.6%, 14.4%, and 9.3%, respectively, demonstrating an acceptable differentiation power. Figure 6 shows the confusion matrix of the testing data from one of the models. Normal tissues were almost correctly classified whereas GBM and PCNSL were partially overlapped with the other. The sensitivity and specificity of GBM were 78.6% and 80.1%, and those of PCNSL were 66.9% and 87.2%, respectively. As a result, an overall accuracy of 79.5% was yielded.

**Fig. 7:**
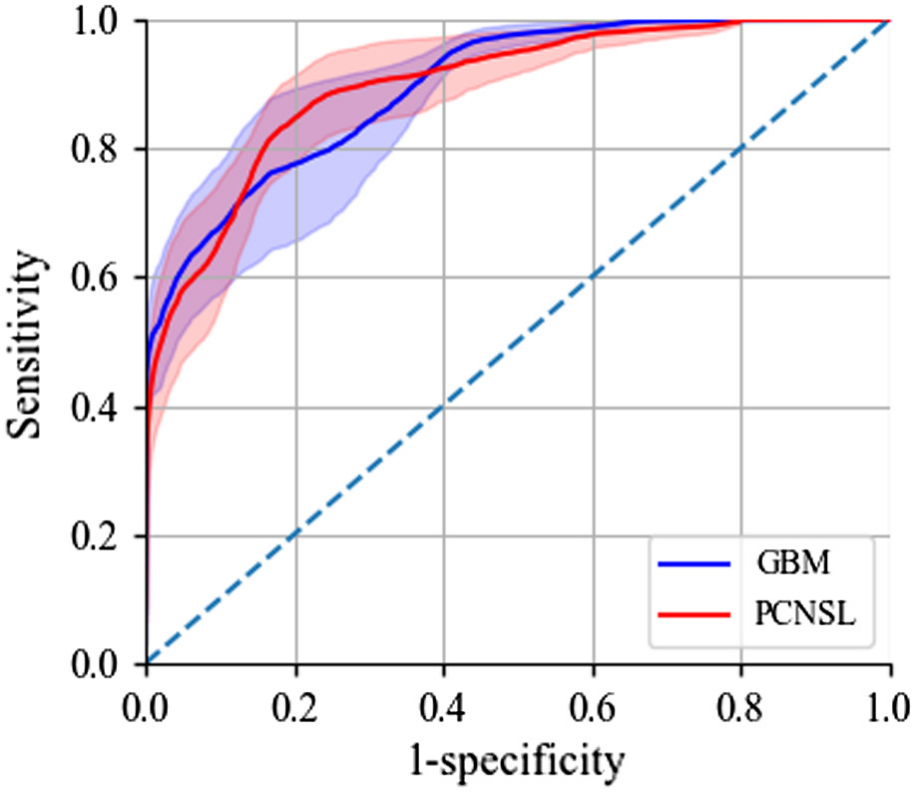
ROC curve of GBM and PCNSL of the attention ResNet model by the testing data. The AUC are 0.896±0.040 and 0.898±0.039, respectively.

To look it more carefully, receiver operating characteristic (ROC) curves of GBM and PCNSL were calculated from the testing data as shown in Fig. 7, and the mean areas under the curves (AUC) were 0.896±0.040 and 0.898±0.039, showing excellent and nearly perfect differentiation powers (0.8≤AUC<0.9) of the targeted tumors. The shadowing wings of the ROC curves represent the mean standard deviation at the corresponding specificities.

### 3.4 Distributions of Data

**Fig. 8:**
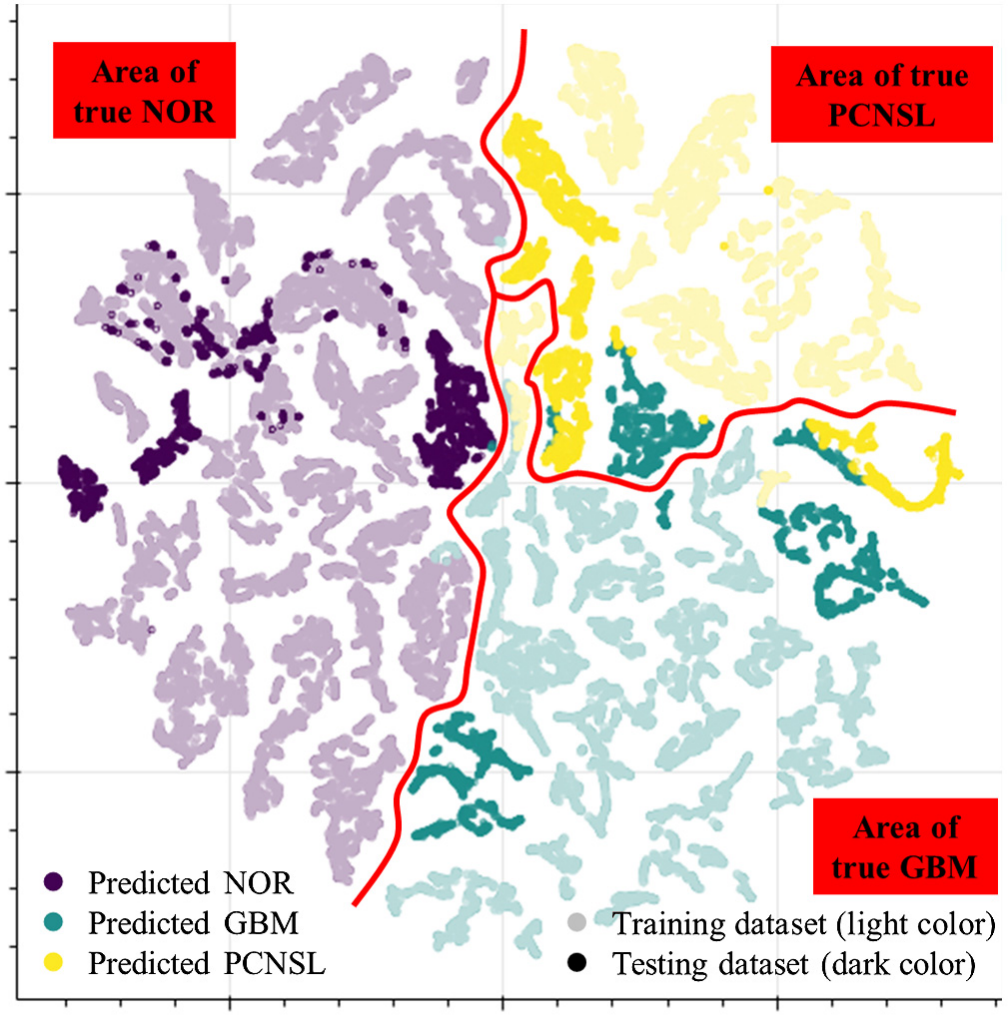
The t-SNE scatter plot of the data points at the last average pooling layer. The colors denoted the predicted label by the model, and the red line showed the true boundary between three categories. Light colors represent training dataset while dark colors represent testing dataset.

We plotted the 2D distribution of the data at the last average pooling layer using t-SNE as shown in Fig. 8. The two axes are the metafeatures of the data, and every data point represents one OCT image and was marked in different colors according to their predicted labels. The training dataset was marked in light colors while the testing dataset was dark colors. The true boundary among normal tissue, GBM, and PCNSL was drawn as the red line, dividing the whole plot into three ground truth categories.

The training dataset was mostly classified correctly while the testing dataset was partially misclassified especially of those distributing at the boundary of GBM and PCNSL. This implies that the characteristics of GBM and PCNSL are similar and are consistent with our findings via the gross visual inspection of OCT images. Of note, the classification could be further improved by slightly adjusting the decision boundary without obvious overfitting.

## 4 Discussions

**Fig. 9:**
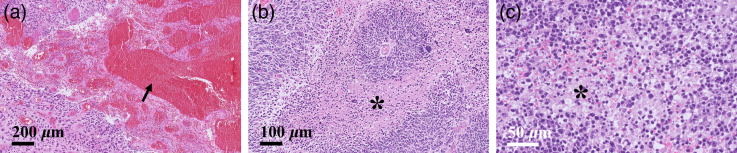
The H&E pathological stained histologies of specimens of the patients. (a) The specimen of the patients diagnosed with GBM exhibited a large area of hemorrhage (arrow) as well as (b) tumoral necrosis (star). On the other hand, (c) the specimen of the patient diagnosed with PCNSL only showed a minor necrotic area (star).

Figure 9 shows the pathological findings of the specimens of the patient the same as those whose OCT images were presented in Figs. 4(b) and 4(c). It was reported that the sample extracted from the patient diagnosed with GBM displayed a large area of hemorrhage and tumoral necrosis. By contrast, the specimen of the patient with PCNSL exhibited only a slight necrotic area. It is known that normal brain tissues display homogeneous and no microstructural appearance, and in the meanwhile, GBM exhibits abnormal microstructure, e.g., the presence of microcysts, calcification, and hemorrhaging in tumoral regions, as well as nonuniform attenuation because of variate cell density caused by hyperplasia or necrosis. On the other hand, although there is still no research regarding PCNSL imaging using OCT, histological findings have shown that the presences of microcysts, calcification, and hemorrhaging in PCNSL are relatively uncommon^40^ are in accordance with our findings in OCT images. This could be an important key to differentiate PCNSL from GBM from a clinical perspective.

In comparison to other quantitative indices, such as attenuation coefficient,^28^ co-channel attenuation, and forward cross-scattering,^26^^,^^30^ our method demonstrated a robust identification power between normal tissues and tumoral tissues with nearly 100% accuracy. Since OCT is capable of differentiating normal tissues from tumoral tissues, eventually this method is also expected to help surgeons with the examination of residual tumor tissues at the end of the surgical excision in the future. Although differentiation between GBM and PCNSL was still to be improved partially due to the limited amount of samples, hopefully, we will be able to achieve high differentiation power based on the current findings of the characteristics of OCT images.

In this research, we showed that tumoral tissues and normal tissues can be distinguished almost perfectly. According to previous reports, GBM tends to display necrotic areas and thus alters the uniformity of attenuation. Nonetheless, radiotherapy and surgical coagulation can also cause tissue necrosis,^27^ and patients treated with radiotherapy were excluded from this study. Also, the porcine brain suffered from no coagulation necrosis. Further studies were warranted to investigate features of tissues from more general occasions. Another concern is that although the cancerous specimens should contain at most infiltrative tissues yet no normal tissues according to the surgical guideline of maximal safe resection, in consideration of a small amount of normal tissue included and appearing in single frames within an OCT volume, the predictive model should be able to identify them rather than simply regard them as the class of a given overall label. As a suggestion for future research, the problem can be addressed by the experimental design of data processing introduced with multiple instance learning, in which negative frames are allowed in a positive OCT volume. Thereby, the prediction accuracy could be further escalated.

Notwithstanding that our method demonstrated high accuracy, the standard deviation of validation accuracy showed only moderate stability. PCNSL itself is a minor population in intracranial neoplasms, and plus normally, patients with PCNSL were not to undergo surgical resection except in the cases of stereotactic biopsy and atypical patterns in MRI. Up to now, we recruited only a few cases of GBM and one case of PCNSL. Given the small number of cases recruited, several techniques including OCT volume-split data separation, early stopping, and data augmentation, have been applied to avoid overfitting. Based on our validation performance, no clear evidence of overfitting was observed. The reason for the drop of accuracy from 97.5% in training data to 80.3% in testing data might also result from underfitting due to insufficient training data size. Although multiple scans were performed to enlarge the amount of OCT images, more cases, especially of PCNSL, were still needed to achieve the model generalization and stability.

The image quality was crucial for training a deep learning model. Despite that we have eliminated images displaying strong reflections from model training, the saturation artifact can still be observed in some images. In addition, due to some small sample sizes, the underlying platform was displayed in OCT images, which might also influence the recognition of characteristics of the model. Fortunately, we ascertained via grad-CAM images that the model focused on meaningful areas instead of on misleading artifacts or objects.

## 5 Conclusions

In this work, we recruited a small number of cases to preliminarily corroborate the feasibility of OCT for GBM and PCNSL identification based on the attention ResNet model in an *ex vivo* experimental design. Once the lesion is predicted as PCNSL using our method, the treatment strategy might be shifted, and the surgeon could decide to close the skull on the spot rather than proceed with the resection; this cannot be brought out by any of the current imaging modalities. Consistent with previous research, normal tissues displayed a homogeneous tissue texture and uniform attenuations while GBM tended to exhibit heterogeneous microstructures and altering attenuations along the transversal direction. By contrast, PCNSL showed no microstructures but slightly variated attenuations, and the underlying pathological cause might be due to the lack of calcification, microcysts, and hemorrhaging in PCNSL tissues. To our knowledge, this was the first time that PCNSL was observed in OCT imaging despite only one case recruited, and the findings of PCNSL in OCT imaging might be an important key to distinguish PCNSL from GBM during the clinical trial.

With the help of the attention ResNet model, an overall testing accuracy of 80.3% was achieved in three categories differentiation, and nearly perfect discrimination was demonstrated between normal tissues and tumoral tissues. This is the first time deep learning implemented in quantitative GBM OCT imaging classification, and the method is robust and promising. We further calculated the ROC curves and AUC of GBM and PCNSL, respectively, and consequently, they demonstrated excellent discrimination powers. Although previous studies have demonstrated visual inspection results of independent investigators, the judgment by bare eye is still subjective, and the result varies among observers and thus is poor in reproducibility. Using CNN in our work provides a quantitative assessment of morphological features of OCT images, which surely enables reproduction of exactly the same results given the same inputs. Furthermore, the diagnostic output can be numerically tweaked by shifting the decisive threshold based on individual clinical needs. By plotting the data distribution at the average pooling layer via t-SNE, we examined the clustering of categories in metafeatures space. We found that all misclassified images were distributed at the boundary of three categories, and that is to say, it is possible to further improve the classification accuracy if enough amount of data for model generalization were available.

In the future, we will recruit more amount of cases to improve the model performance. In consideration of clinical applicability, specimens should be measured *in vivo* or within minutes after resected without formalin fixation as formalin can alter the penetration depth of the light in tissues.^41^ *In vivo* measurement of normal human brain tissues is also one of our future plans to claim a more straightforward conclusion. In addition to the sample preparation, patients treated with radiotherapy should be included in the future recruitment criteria. To probe the underlying classifying mechanism of the model, we are also interested in utilizing other types of CNN architectures, e.g., patch-based classification framework and pixelwise image segmentation, to show the regional diagnostic results in the future. So far, our results show a promising outlook of the combination of OCT and deep learning in GBM and PCNSL identification. We believe this proposed method will assist surgeons intraoperatively and finally improve the prognoses of the patients.

## Data, Materials, and Code Availability

Data underlying the results presented in this paper are not publicly available according to the protection of human research participants.
